# Nutritional iodine status and obesity

**DOI:** 10.1186/s13044-021-00116-y

**Published:** 2021-11-27

**Authors:** Mariacarla Moleti, Maria Di Mauro, Giuseppe Paola, Antonella Olivieri, Francesco Vermiglio

**Affiliations:** 1 Department of Clinical and Experimental Medicine, University of Messina, Messina, Italy; 2 Department of Cardiovascular and Endocrine-Metabolic Diseases and Aging, Italian National Institute of Health, Rome, Italy

**Keywords:** Iodine, Obesity, Body mass index, Thyroid function, Thyroid volume

## Abstract

Iodine is an essential component of the thyroid hormones, thyroxine and triiodothyronine. Its availability strictly depends on iodine content of foods, which may vary from very low to very high. Inadequate iodine intake (deficiency or excess) may affect thyroid function resulting in hypothyroidism or hyperthyroidism. Based on median urinary iodine concentrations, epidemiological criteria have been established for the categorization and monitoring of nutritional iodine status of a population (or subgroups of populations). Additional methods for iodine intake assessment include measurement of thyroid size (by thyroid palpation or ultrasonography) and of biochemical parameters, such as neonatal thyroid stimulating hormone, thyroglobulin and thyroid hormones.

Recent studies carried out in overweight/obese children and adults provide evidence that body mass index (BMI) may significantly influence the above indicators, thus theoretically affecting the epidemiological evaluation of nutritional iodine status in populations.

In this short review, we analyze current knowledge on the effects of overweight and obesity on indicators of adequacy and monitoring of iodine status, namely urinary iodine excretion and thyroid volume and echogenicity.

Data on urinary iodine excretion in overweight/obese children are divergent, as both increased and reduced levels have been reported in overweight/obese children compared to normal-weight controls.

Whether gastrointestinal surgery may affect iodine absorption and lead to iodine deficiency in patients undergoing bariatric surgery has been evaluated in a limited number of studies, which excluded iodine deficiency, thus suggesting that supplements usually recommended after bariatric surgery do not need to include iodine.

Albeit limited, evidence on thyroid volume and obesity is consistent with a direct relationship between thyroid volume and BMI, irrespective of nutritional iodine status. Finally, a higher frequency of thyroid hypoechoic pattern has been described in overweight/obese children. This finding has been recently related to an increased adipocyte infiltration and thyroid parenchyma imbibition mediated by inflammatory cytokines and should be considered when the frequency of thyroid hypoechoic pattern is used as non-invasive marker to indirectly assess thyroid autoimmunity in monitoring Universal Salt Iodization programs. Further studies, specifically addressing the role of schoolchildren body mass index as a factor potentially influencing iodine intake indicators are needed.

## Introduction

Iodine is a key component of the thyroid hormones (THs) thyroxine (T4) and triiodothyroinine (T3), the only iodine-containing molecules in vertebrates. Iodine availability for TH synthesis strictly depends on iodine content of foods, which may vary from very low, as is the case of fruit and most vegetables, to very high, typically found in marine fishes and seaweed [1].

Once ingested, iodide (I^−^) is rapidly and nearly completely absorbed in the stomach and duodenum, whereas iodate (IO^−^) is reduced in the gut and then adsorbed as I^−^. Dietary I^−^ absorption in the gastrointestinal tract occurs mainly via the Na^+^/I-symporter (NIS), which is functionally expressed on the apical surface of enterocytes [2]. Interestingly, evidence has been provided showing that dietary I^−^ is involved in the post-transcriptional regulation of the intestinal NIS, thus contributing to auto-regulate the supply of iodine to the body [3].

Following absorption, I^−^ is rapidly cleared from the circulation by the thyroid gland. The uptake of iodide into the thyroid follicular cells occurs through the NIS located at the basolateral plasma membrane of thyrocytes, which actively transports iodide into the thyroid using the electrochemical gradient generated by the Na/K-ATPase [4]. Overall, I^−^ transport is regulated by the thyroid-stimulating hormone (TSH), which stimulates NIS transcription, half-life and subcellular distribution [5]. In addition, I^−^ regulates its own accumulation and organification, by downregulating NIS and thyroperoxidase (TPO) expression when acutely administered at high doses [6]. Accordingly, the thyroid iodide clearance depends on daily iodine intake and on intrathyroidal iodine stores. In individuals with adequate thyroid iodine stores, the daily amount of iodide taken up by the thyroid gland accounts for about 10–35% of the absorbed dose, the remaining fraction being cleared by the kidney. An additional amount of iodine originating from TH metabolism is also excreted in the urine, so that > 90% of the ingested iodine eventually appears in the urine. Whenever intrathyroidal iodine stores are depleted, the fraction of circulating I^−^ cleared by the thyroid increases up to 65–80% to maintain TH synthesis [7]. This adaptation is mediated by an increase in NIS expression on thyrocytes, which is primary stimulated by TSH at both a transcriptional and post-transcriptional level [8]. Since renal clearance of iodide remains constant also in conditions of nutritional iodine restriction, urinary iodine (UI) determination reflects recent iodine consumption, which is rarely advantageous at individual level as iodine intake may substantially vary from day-to-day [9]. In contrast, measurement of UI concentration in spot urine specimens from representative samples of population provides useful information to estimate dietary iodine intake of target subgroups, such as children, adults, and pregnant/lactating women [10].

Based on median UI concentrations, epidemiological criteria have been established for the categorization and monitoring of nutritional iodine status of a population (or subgroups of populations). Additional methods for iodine intake assessment include measurement of thyroid size (by thyroid palpation or ultrasonography) and of biochemical parameters, such as neonatal TSH, Thyroglobulin (Tg) and THs [10].

Recent studies carried out in overweight/obese children and adults provide evidence that body mass index (BMI) may significantly influence the above indicators, thus theoretically affecting the epidemiological evaluation of nutritional iodine status in populations. In order to address this topic, we carried out this narrative review to analyze current knowledge on the effects of overweight and obesity on indicators of adequacy and monitoring of iodine status, namely UI excretion and thyroid volume and echogenicity.

### Urinary iodine and obesity

The relationship between UI concentrations and obesity has been explored in both schoolchildren and adults, as well as in longitudinal studies involving obese subjects prior to and after bariatric surgery (Table 1).

**Table 1:** Summary of the studies reporting data on UIC in overweight/obesity

First Author *(ref)*	Year	Country	Study group *(n)*	Study group iodine status	Main finding(s)
Garcia-Solis P [11]	2013	Mexico	Schoolchildren *(n = 1544)*	more than adequate	+ve correlation between UIC and OW/OB
de Oliveira Campos R [12]	2016	Brazil	Schoolchildren *(n = 1419)*	sufficient	no significant correlation between UIC and BMI; reduced risk of excessive iodine intake in OW/OB children
De Angelis S [13]	2021	Italy	Schoolchildren *(n = 1595)*	sufficient/ mildly deficient	-ve correlation between UIC and OW/OB in iodine sufficient children; no significant correlation between UIC and OW/OB in mildly iodine deficient children
Farebrother J [14]	2021	UK	Obese pregnant women *(n = 954)*	inadequate	sub-optimal iodine status amongst multi-ethnic pregnant women with obesity
Michalaki M [15]	2014	Greece	morbidly obese adults before and after bariatric surgery *(n = 35)*	sufficient	UIC unaffected by bariatric surgery and weight loss
Lecube A [16]	2015	Spain	morbidly obese women *(n = 90)*	mildly deficient	UIC negatively correlated with BMI
Manousou S [17]	2018	Sweden	obese adults with or without bariatric surgery *(n = 4047)*	sufficient	lower, though sufficient, iodine status in bariatric groups than in obese controls

*+ve* positive, *−ve* negative, *UIC* urinary iodine concentrations, *OW* overweight, *OB* obesity

#### Urinary iodine and obesity in schoolchildren and in pregnant women

The very first observation of a direct relationship between UI concentration (UIC) and weight dates back to 2013, when Garcia-Solis and co-workers carried out an epidemiological study aimed at estimating UIC in primary school and at correlating it with global nutrition indicators and social gap index (SGI) [11]. Fifty elementary schools from 10 municipalities in the State of Queretaro (Mexico) were screened and an overall of 1544 schoolchildren were enrolled in the study. Median UIC obtained in the 50 survey sites indicated a more than adequate iodine intake (297 μg/L), with dietary daily iodine consumption resulting more than adequate (19/44) or even excessive (25/44) in most of the schools. Medians of UIC per school were found to be positively correlated with medians of BMI standard deviation score (SDS), height SDS, and overweight and obesity prevalence, and negatively correlated with stunting prevalence and SGI. In the Authors’ interpretation, unwholesome dietary habits in this subset of population, in particular a wide consumption of snack food rich in energy and iodized salt, might explain the observed association between iodine excess and unhealthy weight [11].

A large cross-sectional study was carried out as part of a national project of salt iodization implemented in Bahia, a northeastern region of Brazil with precarious socio-economic and demographic conditions. In this study 1419 schoolchildren aged 6–14 years were recruited, and anthropometric parameters, UIC, and TSH were measured. Median UIC (221.6 μg/L) indicated a condition of iodine sufficiency and the majority of schoolchildren (80.9%) fell within the normal range for UIC (100–299 μg/L). Nonetheless, in 12.3% schoolchildren urinary iodide concentration were < 100 μg /L and 9.4% presented excessive iodine intake (EII, UIC > 300 μg/L). In contrast to the previous study, no significant correlation between UIC and BMI was found and, even more telling, the risk of EII was estimated to be reduced of 36% in overweight/obese schoolchildren [12]. Unfortunately, the Authors do not provide any explanation for this latter finding, and whether overweight/obesity protected against EII because of different dietary habits and/or socioeconomic factors remains unclear.

Finally, a recent study carried out in Italy and involving schoolchildren from iodine repleted and marginally iodine deficient areas within the same country showed a different distribution of UIC values in normal-weight and overweight/obese children according to background iodine status. In particular, in iodine sufficient children median UIC was significantly lower in obese than in normal-weight children (102 μg/L vs 135 μg/L), whereas no significant difference was observed between the latter and overweight children (135 μg/L vs 118 μg/L). By contrast, in mildly iodine deficient children UIC was similar in all the above BMI categories, although obese boys had a median UIC value indicative of iodine sufficiency (113 μg/L) and obese girls of iodine deficiency (84 μg/L) [13]. Whether these findings were due to a true effect of BMI or to confounding factors, such as severity of obesity or social and economic factors, could not be ascertained in this study, since no information was collected on education and economic status of children’s parents.

Iodine nutritional status amongst multi-ethnic pregnant women with obesity from inner-city areas in the UK was recently assessed by Farebrother and coll [14]., as part of the UK Pregnancies Better Eating and Activity Trial (UPBEAT). Iodine and creatinine concentrations were measured in spot urine samples in the second trimester (15th–18th weeks) of gestation in 954 obese (BMI > 30 kg/m^2^) pregnant women, and relationship between iodine nutrition and birthweight determined using linear and logistic regression models. An UI/creatinine (UI/Cr) < 150 μg/g was found in 70% of women, who also had a trend to deliver infants with a lower birthweight. The Authors conclude that the iodine nutrition intake of this cohort of UK pregnant women with obesity was suboptimal and that inadequate iodine status was associated with lower birthweight. As the Authors state, generalizability of these results is limited because of a lack of control group of normal-weight pregnant women [14].

#### Urinary iodine and obesity in bariatric surgery

Whether gastrointestinal surgery may affect iodine absorption and lead to iodine deficiency in patients undergoing such surgical procedures has been evaluated in a limited number of studies. Actually, the very first observation of a direct relationship between UIC and total gastrectomy dates back to 1964, when Harden and Adams first reported iodine deficiency in 6 of 8 patients who underwent total gastrectomy, likely as a result of iodine malabsorption [18]. Fifty years later, daily iodine intake was also estimated in severely obese Greek patients before and after bariatric surgery by Michalaki et al. [15]. Thirty-five severely obese patients (obese group) with a BMI of 51.3 ± 8.3 kg/m^2^ were studied before, 3, and 6 months after bariatric surgery and compared with a control group of 35 subjects living in the same house and following similar diet. UIC at baseline was similar in obese and control group (median 129.5 μg/L vs 138.9 μg/L). In the obese group, UIC transiently increased 3 months after the operation, but returned to baseline levels 6-months post-surgery. Based on these data, UIC is not affected by malabsorptive bariatric surgery, although all stomach, duodenum, and a substantial part of jejunum are bypassed, thus suggesting that iodine is sufficiently absorbed along the remaining gastrointestinal tract.

In contrast to the above results are those by Lecube and coworkers [16]. Baseline urinary iodine levels of morbidly obese women (*n* = 90) were compared with those of either 90 women with at least 18 months follow-up after bariatric surgery or 45 healthy non-obese women recruited between family members from the two previous groups. In this study, urinary iodine was measured using the inductively coupled plasma mass spectrometry (ICP-MS) method and expressed as the iodine to creatinine (UI/Cr, μg/g) ratio. UI/Cr was significantly lower in obese women (BMI 43.6 ± 5.0 kg/m^2^, UI/Cr 96.6 μg/g) than in obese postsurgery women (BMI 31.4 ± 6.2 kg/m^2^, UI/Cr 131.9 μg/g) and in non-obese controls (BMI 24.3 ± 3.9 kg/m^2^, UI/Cr 173.3 μg/g). Accordingly, the proportion of women with adequate iodine status progressively increased in the above groups (46.6%, 74,5, 83.3%, respectively). Multiple linear regression analyses showed BMI to be independently associated with UIC, thus suggesting that obesity is an independent risk factor for iodine deficiency, at least in women [16].

The largest and longest prospective study on iodine status after bariatric surgery was carried out in Sweden by Manousou and co-workers [17]. The Swedish Obese Subjects (SOS) was a prospective, non-randomized study of 4047 obese women, recruited from 1987 to 2001, who decided to undergo bariatric surgery (*n* = 2010) or non-surgical treatment (*n* = 2037) (SOS groups). Twenty-four hours urinary iodine excretion (24 h-UIE) was compared at baseline and, after 2 and 10 years following bariatric surgery in the two groups. Based on the surgical approach, patients were further stratified into vertical banded gastroplasty (VBG *n* = 188) and gastric by-pass (GBP *n* = 188) groups. These patients were compared with a random population-based sample from the World Health Organization MONItoring of trends and determinants for CArdiovascular disease (WHO MONICA, *n* = 412 subjects). At baseline, median 24 h-UIE was higher in GBP patients, VBG patients and OB controls than in normal-weight MONICA controls (214 μg/24 h, 201 μg/24 h, 203 μg/24 h vs 137 μg/24 h). At 10 years, 24 h-UIE in GBP patients (161 μg/24-h) and VBG patients (149 μg/24-h) was lower compared with baseline and OB-controls (189 μg/24-h), but similar to 24 h-UIE in MONICA controls (137 μg/24-h). The 10-year UIE was similar in GBP-patients and OB-controls, but higher in VBG-patients. The Authors concluded that, despite the fact that both groups (GBP and VBG-patients) had lower iodine nutritional status than OB-controls, they did not suffer from iodine deficiency, thus suggesting that supplements usually recommended after bariatric surgery do not need to include iodine, at least in iodine sufficient countries [17].

### Thyroid volume and obesity

Thyroid size is a valuable indicator in baseline assessments of iodine intake, but much less so in monitoring programs. Actually, this measure does not reflect the current intake of iodine in the diet, as thyroid size may remain unchanged for months or years after correction of iodine deficiency [10]. The target groups of goiter surveys are usually school-age children (6–12 years), but also surveillance of pregnant and lactating women and women of childbearing age (15–44 years) is paramount because these subgroups are especially sensitive to even marginal iodine deficiency.

Thyroid volume is preferentially assessed by ultrasonography, which provides a more precise measurement of thyroid volume than palpation. To define severity of iodine deficiency, the following total goiter rates in schoolchildren are used: < 5%, iodine sufficiency; 5.0–19.9%, mild deficiency; 20.0–29.9%, moderate deficiency; and > 30%, severe deficiency [10]. Importantly, results of ultrasonography need to be interpreted according to reference criteria, taking into account age, gender, along with body surface area (BSA).

A positive correlation between thyroid gland size and body weight was first reported almost 40 years ago by Hegedüs and colleagues, who also found no significant differences in the thyroid volume/body weight ratio between males and females in an unselected population of 271 healthy normal-weight subjects (age 13–91 years) from a moderate iodine deficient area [19]. Similarly, thyroid volume has been shown to increase with the increasing of anthropometric parameters, either in adults [20–25] or in children [13, 26–29], irrespective of the nutritional iodine status.

Concerning the relationship between obesity and thyroid volume, data in the literature are rather conflicting (Table 2).

**Table 2:** Summary of the studies reporting data on thyroid volume in overweight/obesity

First Author *(ref)*	Year	Country	Study group *(n)*	Study group iodine status	Main finding(s)
Sari R [30]	2003	Turkey	Pre-menopausal obese women *(n = 98)*	NA	higher TV and goiter prevalence in obese women; not significant change in TV for > 10% weight loss
Eray E [31]	2011	Turkey	Pre-menopausal obese women *(n = 98)*	sufficient	-ve relationship between TV and UIC; no significant association between TV and weight loss
Méndez-Villa L [32]	2016	Mexico	Schoolchildren *(n = 673)*	more than adequate	higher iodine intake and TV in OW than in AW children
De Angelis S [13]	2021	Italy	Schoolchildren *(n = 1595)*	sufficient/ mildly deficient	TV distribution in AW children significantly lower in comparison to TV distribution in OB children, irrespective of the iodine status.

*NA* not assessed, *TV* thyroid volume, *−ve* negative, *AW* adequate weight, *OW* overweight, *OB* obese

In a population study involving 98 premenopausal obese (BMI > 30 kg/m^2^) and 31 age-matched non-obese (BMI < 25 kg/m^2^) women from a mild/moderate iodine-deficient area, higher goiter prevalence (24.5% vs 12.9%) and increased thyroid volume (15.2 ± 5.3 mL vs 12.2 ± 3.3 mL) was found in obese women. In addition, compared to baseline, a > 10% weight loss after 6 months of obesity treatment resulted in a significant decrease in thyroid volume, and this change was mirrored by a significant reduction in TSH concentrations over the same time span [30]. In this study, however, urinary iodine was not measured, so that the role of iodine nutrition could not be evaluated. In a subsequent study, however, the same research group, in addition to the expected positive correlation between thyroid volume and BMI, TSH and leptin levels in obese women, reported of a negative relationship between thyroid volume and urinary iodine levels. Nonetheless, no significant association was observed between thyroid volume and weight loss, thus suggesting that iodine status may play an important role for increased thyroid volume in obese women, while not affecting changes in thyroid volume during weight loss [31].

In a cross-sectional study performed during 2012–2013, thyroid volume was measured by ultrasound in 673 schoolchildren from Queretaro, Mexico. The prevalence of overweight/obesity in the studied population was as high as 47.3%, and median UI concentrations above 300 μg/L, thus indicating a high iodine intake. Overweight and obese girls at the age of 8, 10 and 12 years and boys at the age of 6 years showed higher thyroid volume than their corresponding normal weight groups, and in the 6–8-year-old group, obese schoolchildren had higher iodine intake than normal-weight children. According to the Authors, the differences in thyroid volume between normal-weight and overweight/obese schoolchildren could be mainly explained by the higher BSA in the latter, rather than by a higher iodine intake from table salt and processed foods [32].

Very recently, data on the effects of overweight and obesity on thyroid volume in schoolchildren were provided [13]. The study population was part of a nationwide survey carried out by the Italian National Observatory for Iodine Prophylaxis and included 1281 children aged 11–13 years residing in iodine-sufficient regions (IS-group, median UIC 129 μg/L) and 314 age-matched children from a mildly iodine deficient area (ID-group, median UIC 89 μg/L) within the same country (Italy) [33]. Based on the Italian Cross-Sectional Growth Charts for Height, Weight and BMI [34], the recruited children were classified as adequate weight (AW), overweight (OW), or obese (OB). UIC was measured by using inductively coupled plasma mass spectrometry and thyroid ultrasound was performed to assess both thyroid volume and echogenicity. The results of this study show that iodine intake significantly affected thyroid volume, in that it was significantly higher in ID than IS children at any age. However, when thyroid volume values in AW, OW, and OB children were compared, the distribution of thyroid volume in AW children was significantly lower in comparison to the distribution of thyroid volume in OB children, irrespective of the iodine status. In addition, among iodine-sufficient children the distribution of thyroid volume in AW children was significantly lower than the distributions observed in OW children. Thus, the results from this study demonstrate that BMI may be a confounding factor in monitoring iodine nutritional status of populations, as evaluation of the latter may be biased by the recruitment of overweight and obese subjects in the target sample [13].

Interestingly, this study also documented a significantly higher frequency of thyroid hypoecogenicity among obese children, either in iodine sufficiency or deficiency. This result is consistent with previous data by Radetti et al and demonstrating a high prevalence in obese children of ultrasonographic thyroid changes that were superimposable onto that commonly observed in patients with the classical form of autoimmune thyroiditis, though not apparently sustained by autoimmunity [35]. In a subsequent study, the same research group also reported of a significant improvement in thyroid structure (quantified according to a pediatric scoring system [36]), in children with obesity who followed a successful weight-management program [37], thus reinforcing the hypothesis that the observed structural changes are related to cytokines and other inflammatory markers produced by adipose tissue.

In a retrospective analysis, Rotondi et al. compared thyroid US scans showing a hypoechoic pattern of the thyroid from 105 morbid obese patients (BMI > 40 kg/m^2^) and 105 non-obese patients (BMI ≤ 30 kg/m^2^). Overall, there were 2/105 (1.9%) patients in the non-obese group but 68/105 (64.8%) patients in the obese group who showed a hypoechoic pattern of the thyroid at US not accompanied by thyroid abnormality, as assessed by a complete thyroid work-up, including clinical examination, thyroid morphology, hormones, and autoantibodies measurements [38]. This evidence thus suggests that also in adults a thyroid hypoechoic pattern at ultrasonography may be misleading when patients with morbid obesity are considered. A possible explanation for these data comes from a very recent histological study aimed at evaluating whether the thyroid tissue of subjects with overweight or obesity presented any differences in histology and in gene expression profiling, compared to the thyroid tissue of normal-weight individuals. Included in the study were 30 normal-weight subjects, 34 overweight subjects and 34 subjects, who underwent thyroidectomy for benign (TIR 2) or undetermined (TIR 3) nodules and no evidence of thyroid autoimmunity. The rate of adipose cells progressively increased from normal-weight (40%) to overweight (52.9%), and obese (73.5%) subjects, and the number of CD3+ and CD8+ lymphocytes was higher in patients with overweight and obesity than in normal-weight subjects [39].

Taken together the above evidence indicates that in subjects with obesity the enlargement of thyroid gland may be explained by adipocyte and lymphocyte infiltration, which, in conjunction with thyroid parenchyma imbibition mediated by inflammatory cytokines, may contribute to the appearance of a hypo-echogenic pattern unrelated to thyroid autoimmunity.

## Conclusions

An adequate iodine nutritional status is essential for thyroid hormone synthesis. When the dietary iodine intake is insufficient, both T4 and T3 biosynthesis may be compromised, and hypothyroidism may occur. The clinical consequences of this condition depend on the severity, timing and length of iodine deprivation and result in a wide spectrum of clinical manifestations, collectively known as Iodine Deficiency Disorders (IDD) [40, 41]. On the other hand, a dietary iodine intake above the recommended threshold may be responsible for the occurrence of hyperthyroidism, hypothyroidism, goiter, and/or thyroid autoimmunity. Specific subsets of population, namely pregnant women and children are especially vulnerable to both inadequate and excessive iodine intakes, as thyroid function disruption during these stages of life may irreversibly impact growth and intellectual development [42].

Because of the severity of several health consequences of inadequate iodine nutrition, initiatives to guarantee adequate population iodine intakes and maintenance of levels within an optimal range have long been implemented. Assessment of iodine nutrition at the population level mainly relies on determination of salt iodine levels, estimation of household coverage of adequately iodized salt, and UI and thyroid volume measurement in schoolchildren. Concerning the last two indicators, UI is a good marker of very recent dietary iodine intake, whereas thyroid volume assessment by thyroid ultrasound in schoolchildren is an indicator of a long-lasting iodine intake in a population. In addition, the assessment of thyroid hypoechogenicity on US has been also proposed as an indirect indicator of iodine-induced thyroid autoimmunity in epidemiological surveys finalized to monitor iodine prophylaxis programs [43, 44].

As stated above, children are the target population of most iodine monitoring programs, and evidence is accumulating which suggests that overweight or obesity in this subset of population may significantly influence some of the indicators of iodine intake. Since childhood obesity is a growing health problem in developing and developed countries [45], the question rises whether inclusion of overweight/obese children in epidemiological surveys may (and to what extent) affect the results obtained. Actually, data on UI excretion in overweight/obese children are divergent, as both increased and reduced UI levels are reported in overweight/obese children compared to normal-weight controls.

Albeit limited, evidence on thyroid volume and obesity is consistent with a direct relationship between thyroid volume and BMI, irrespective of nutritional iodine status. Finally, a higher frequency of thyroid hypoechoic pattern has been described in overweight/obese children. This finding has been related to an increased adipocyte and lymphocyte infiltration and thyroid parenchyma imbibition mediated by inflammatory cytokines. BMI should be therefore considered when the frequency of thyroid hypoechoic pattern is used as non-invasive marker to indirectly assess thyroid autoimmunity in monitoring USI programs.

In conclusion, though limited by the narrative design, this review highlights that a too small number of studies exploring the effects of obesity and overweight on indicators of iodine intake is so far available to draw any firm conclusion on this topic. Further studies specifically addressing the role of schoolchildren BMI as a factor potentially influencing iodine intake indicators are therefore needed to make monitoring of iodine prophylaxis programs more accurate and representative.

## Data Availability

Not applicable.
